# Hybrid Fiber-Optic Sensing Integrating Brillouin Optical Time-Domain Analysis and Fiber Bragg Grating for Long-Range Two-Parameter Measurement †

**DOI:** 10.3390/s21124224

**Published:** 2021-06-20

**Authors:** Shien-Kuei Liaw, Chi-Wen Liao, Meng-Hsuan Tsai, Dong-Chang Li, Shu-Ming Yang, Zhu-Yong Xia, Chien-Hung Yeh, Wen-Fung Liu

**Affiliations:** 1 Department of Electronics and Computer Engineering, National Taiwan University of Science and Technology, Taipei 10671, Taiwan; tsaimh00101000@gmail.com (M.-H.T.); d10604801@gapps.ntust.edu.tw (D.-C.L.); M10602312@mail.ntust.edu.tw (S.-M.Y.); vu845jm6359@gmail.com (Z.-Y.X.); 2 Energy and Environment Research Laboratories, Industrial Technology Research Institute, Hsinchu 31048, Taiwan; cwliao@itri.org.tw; 3 Department of Photonics, Feng Chia University, Taichung 40724, Taiwan; yehch@fcu.edu.tw (C.-H.Y.); wfliu@fcu.edu.tw (W.-F.L.)

**Keywords:** Brillouin optical time-domain analysis, fiber Bragg grating, distributed fiber sensing, point to point, hybrid sensing

## Abstract

Distributed fiber sensing (DFS) can provide real-time signals and warnings. The entire length of fiber optic cable can act as a sensing element, but the accuracy is sometimes limited. On the other hand, point-to-point fiber sensing (PPFS) is usually implemented using one or more fiber Bragg gratings (FBGs) at specific positions along with the fiber for the monitoring of specific parameters (temperature, strain, pressure, and so on). However, the cost becomes expensive when the number of FBGs increases. A hybrid fiber sensing scheme is thus proposed, combining the advantages of DFS and PPFS. It is based on a Brillouin optical time-domain analysis (BOTDA) fiber system with additional FBGs embedded at certain positions where it is necessary to detect specific parameters. The hybrid fiber sensing system has the advantages of full sensing coverage at essential locations that need to be carefully monitored. In our work, the test results showed that the proposed system could achieve a sensing distance of 16 km with the single-mode fiber with a 2 m spatial resolution. For FBG parameter measurements, the temperature variation was 52 °C, from 25 °C to 77 °C, with a temperature sensitivity of 23 pm/°C, and the strain was from 0 to 400 µε, with a strain sensitivity of 0.975 pm/µε, respectively, using two FBGs.

## 1. Introduction

In 1966, Kuen Kao demonstrated that fiberglass could be used as a medium for transmitting light and proposed that if the decay per kilometer were less than 20 dB, optical communication would be possible [1], bringing the communications industry to the next generation. As a result, optical fiber research was invested during the 1970s, aiming to reduce optical fiber attenuation, achieve long-distance transmission, and begin commercial use. Simultaneously, optical fiber sensing technology also started to develop, which could measure physical quantities such as temperature, pressure, stress, strain, and acceleration.

Compared to traditional electronic sensors, the advantages of fiber optic sensors include high sensitivity, long transmission distance, low attenuation rate, small size, anti-corrosion properties, anti-electromagnetic interference properties, high-temperature tolerance, and long sustainability [1,2]. Optical fiber sensing can be divided into two categories: point-by-point fiber sensing and distributed fiber sensing. Usually, fiber Bragg gratings (FBGs) are used for point-to-point sensing. This is based on inserting one or more FBGs at certain places and fiber links for parameters (temperature, strain, pressure, and so on) monitoring. However, the number of FBGs required is significant if multiple detection points are necessary. In addition, it may result in substantial total insertion loss and is expensive.

Moreover, in FBGs, there is no sensing ability or fiber link other than at those locations. Distributed fiber sensing is based on the Brillouin scattering effect along with the fiber link. The entire fiber point is the sensing element, but the accuracy is limited. In other words, there is a displacement error between the actual point and the predicted point in a distributed fiber sensing system. There are two typical applications of distributed fiber optic sensing: Brillouin optical correlation domain analysis (BOCDA) [3] and Brillouin optical time-domain analysis (BOTDA) [4]. These two methods rely on analyzing subtle drifts of Brillouin frequency to achieve higher spatial resolution and a more extended probing range for temperature and strain sensing. Recent advances in Brillouin OTDA have achieved sensing ranges of up to ten kilometers with a displacement error of less than one meter. Although the resolution of BOCDA is more accurate [5], the probing range is much shorter than BOTDA, which limits the applicability are sensing distances longer than a kilometer. Therefore, BOTDA is preferred for carbon storage applications.

Brillouin optical time-domain analysis (BOTDA) was first proposed by Horiguchi’s group in 1989, who realized a system with 1.2 km SMF with 10 m resolution [6]. Later, Xiaoyi’s group successfully demonstrated a system with a 10 m resolution over a 50 km distance [7]. Carbon capture and storage is one of the practical options for reducing greenhouse gas emissions. Carbon dioxide is captured from large stationary emitters and transported to the storage site, injected into deep geological formations. The operation of CO_2_ injection could induce fluctuations in reservoir pressure and temperature, which would provide information on CO_2_ plume movement. Improper injection strategy and pressure control may damage the wellbore and influence reservoir integrity, increasing overall project risk. Therefore, it is necessary to deploy equipment for pressure and temperature monitoring. Downhole temperature and pressure gauges are commonly used for geological storage monitoring. Although they provide accurate measurements, the spatial coverage is limited. BOTDA, which can act as an array of sensors, can be deployed to overcome this limitation and provide real-time information on reservoir temperature and pressure.. Y. Sun et al. presented distributed fiber optic sensing for monitoring geological CO_2_ sequestration [8]. It possesses excellent potential to sense minor disturbances in the deep subsurface. A. Zrelli et al. monitored temperature and pressure in disaster environments using Brillouin optical sensors [9].

Distributed optical fiber sensing can provide real-time signals and warnings, helping preserve the safety of storage sites. However, there are still some difficulties in determining the exact locations at which important parameters fluctuate. On the other hand, point-by-point fiber sensing is capable of accurate positioning. Therefore, we propose the possibility of combining the point-by-point fiber sensing with distributed fiber sensing within the same system by placing several FBGs in a BOTDA sensing system. This would offer the advantages of providing sensing ability along all of the fiber links (distributed), while the most dangerous/essential points could be applied with pre-located FBGs to achieve precise measurements of variations in these parameters. We named the sensing scheme a “hybrid sensing system.” However, this methodology has seldom been seen in prior works. Only three other prior works have demonstrated BOTDA/FBG sensing. T. Nannipieri et al. mentioned a hybrid BOTDA/FBG sensor for discrete dynamic and distributed static strain/temperature measurements [10]. J. Li et al. presented an FBG-based positioning method for BOTDA sensing [11]. Zhou et al. demonstrated large-scale BOTDA and localized FBGs [12]. In general, these papers focused only on the measurement of the strain parameter. In this paper, in addition to the strain parameter, the thermal parameter is also discussed in detail for long BOTDA fiber sensing systems. Moreover, D. Ba et al. demonstrated a temperature-insensitive shape sensor based on BOTDA with a short distance of 10 cm [13]. H. Iribas et al. studied the effects of pump pulse extinction ratio (ER) in a BOTDA system. Their experiments confirmed that a long-range BOTDA deploying a 26-dB ER EOM and an optical amplifier could exhibit a Brillouin frequency shift error higher than 1 MHz, even with minimal probe power [14]. W. Lin et al. demonstrated Brillouin gain bandwidth reduction in a BOTDA system, obtaining more precise temperature/strain measurements [15]. Arnaldo G. Leal-Junior et al. proposed a technique for designing and optimizing springs for specific operating conditions or design requirements, meeting the instrumentation requirements of soft robots and compliant structures [16]. Daniele Tosi et al. extended the optical backscatter reflectometry method from a single fiber to multiple fibers using a high-scattering optical network to scan multiple fiber segments simultaneously, providing a reference for the use of SLMux in biomedical devices [17]. Arnaldo G.Leal-Junior et al. proposed a multiplexing technology for sensors based on changes in polymer optical fiber strength. Tests have also been carried out in multi-parameter applications. This technology has guided the design of sensor arrays [18]. V.V. Zaharov et al. demonstrated that Karhunen–Loève treatment could be used as a powerful data de-noising tool in the proposed sensing applications [19].

This paper aims to design and assemble a sensing system to analyze the Brillouin frequency drift caused by fluctuations in temperature and strain. Then, the paper proposes and demonstrates such a hybrid sensing system by putting two FBGs at two locations for temperature and strain measurement. A sensing range of up to 16 km was implemented for measurement. The Brillouin frequency drift induced by temperature and strain and the FBG shift was recorded and presented. Parts of the paper were published as part of the 25th OptoElectronics and Communications Conference (OECC 2020) [20]. In this paper, further analyses and detailed illustrations of the works are provided.

## 2. Materials and Methods

Brillouin scattering consists of spontaneous Brillouin scattering and stimulated Brillouin scattering. The critical value is referred to as the stimulated Brillouin scattering (SBS) threshold. When the incident pump laser energy is greater than the stimulated Brillouin threshold, it is called SBS. If the pump laser energy is lower than the stimulated Brillouin threshold, it is spontaneous Brillouin scattering.

### 2.1. Spontaneous Brillouin Scattering

When light is transmitted in optical fiber, sound waves are produced due to the Brownian motion of optical fiber molecules. The pressure generated by sound waves causes periodic refractive index changes in the optical fiber material. This phenomenon can be regarded as Brillouin optical grating, moving in the direction of the incident light in the optical fiber, producing a random scattering effect on the transmitted light wave. The interaction between the light and sound waves causes part of the incident light to backscatter, and the movement of the sound wave leads to the Doppler effect, resulting in a Brillouin shift. The frequency of the incident light after the frequency reduction is the frequency of the scattered light, referred to as the Brillouin frequency $\nu_{B}$. The relationship between the Brillouin frequency shift and the acoustic wave is as shown in Equation (1).

(1) $$\nu_{B}=\frac{2n\cdot \nu_{A}}{\lambda_{p}}$$

where n is the refractive index of the medium, $\nu_{A}$ is the group velocity of the sound wave, and $\lambda_{p}$ is the pump laser wavelength. Therefore, the Brillouin frequency is determined by the sound wave’s group velocity, the wavelength of the pump laser, and the refractive index of the medium. Taking the standard single-mode fiber as an example, when the pump laser wavelength is 1550 nm, the calculated Brillouin frequency is about 11 GHz. The Brillouin frequency depends on the refractive index n of the optical fiber and the sound wave’s group velocity $\nu_{A}$. The group velocity of the transmitted sound wave in the solid is shown in Equation (2).

(2) $$\nu_{A}=\sqrt{\frac{K}{\rho }}$$

where K is the bulk modulus of silicon dioxide, and *ρ* is the average density in the optical fiber. The density varies with ambient temperature or strain. Therefore, Brillouin scattering can be applied to probe temperature, strain, and other parameters based on this characteristic.

### 2.2. Stimulated Brillouin Scattering

When the pump laser power is greater than the SBS threshold, the spontaneous Brillouin scattering will be transformed into stimulated Brillouin scattering. The interference between the backscattered Stokes light and the pulse pump laser results in an electrostriction effect in the fiber. The coherent elastic sound field generated by the electrostriction effect excites more Brillouin scattering light, and at the same time, the sound field excited by the scattering light becomes more robust. As a result, a greater degree of power will be obtained in this scattering light during this repeated process, which is referred to as stimulated Brillouin scattering—the interference relationship between the incident light and scattered light and sound wave.

The power threshold formula for stimulated Brillouin scattering is shown in Equation (3).

(3) $$P_{SBS}=\frac{21A_{eff}}{g_{B}L_{eff}}$$

where $A_{eff}$ is the effective optical fiber area, $L_{eff}$ is the effective length of optical fiber, and $g_{B}$ is the Brillouin gain coefficient.

The incident laser pulse power must be less than the stimulated Brillouin scattering power threshold; otherwise, the incident laser pulse will excite the detection signal’s acoustic wave. This phenomenon means that if the power of the pump laser pulse is greater than the stimulated Brillouin scattering power threshold, the frequency difference between the pump laser and the detection light will fall outside of the Brillouin gain bandwidth of the fiber. Thus, the Brillouin scattering signal can still be measured, but the measurement accuracy will be reduced.

When the pulse width of the pump laser is much greater than the phonon lifetime in the fiber (10 ns), the stimulated Brillouin scattering can be regarded as stationary, not changing with time, and thus approximating the pump laser acoustic Stokes field. On the other hand, the amplitudes of the three-wave fields (i.e., the pump laser field, the acoustic wave, and the Stokes field) do not change with time; a phenomenon called the stimulated Brillouin scattering steady-state theory. If the steady-state analysis method is adopted, the simplified formula is as follows:

(1) The pulse width of the pump laser is much larger than the phonon lifetime in the fiber, and all the time terms in the coupled wave equation are ignored and eliminated.
(2) The frequency of the pump pulse is approximately equal to that of the backscattered Stokes light.
(3) The attenuation coefficients of the pump pulse light and the backscattered Stokes light in fiber are the same.

**Figure 1:**
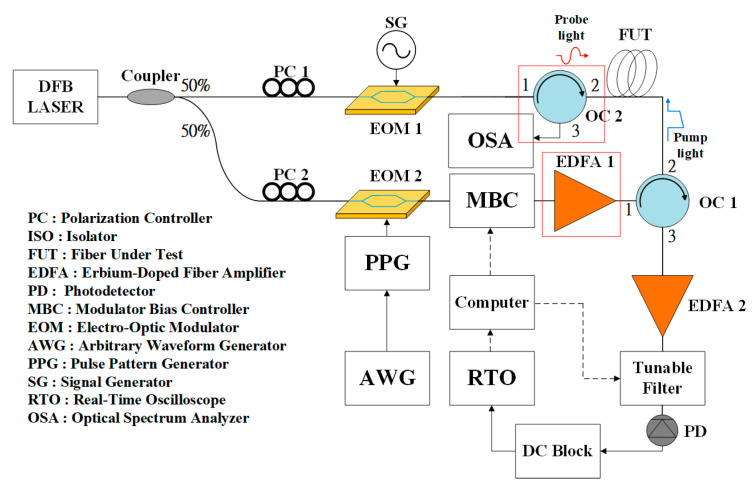
Hybrid system combining fiber grating and BOTDA system.

According to these three assumptions, the simplified SBS steady-state coupled wave equation can be expressed as follows:

(4) $$\;\frac{dI_{p}}{dz}=-g_{B}I_{p}I_{s}-\alpha I_{p}$$

(5) $$\frac{dI_{s}}{dz}=-g_{B}I_{p}I_{s}+\alpha I_{s}$$

where $I_{p}$ is the intensity of the pump pulse light, $I_{s}$ is the Stokes intensity, α is the attenuation coefficient of the fiber, $g_{B}$ is the gain coefficient, L is the length of the fiber to be measured, z is the position in the fiber to be measured, and 0 ≤ z ≤ L. This formula can be used to calculate the light intensity and Brillouin gain. By measuring the strain and temperature changes of the fiber grating, the measured value can be compared with the value measured by the Brillouin optical time-domain analysis system. In Figure 1, an optical circulator is added to the connection end of the optical fiber to be tested, and the Photodetector and the optical isolator are removed. Port 3 of the optical circulator is connected to an optical spectrum analyzer to observe the penetration spectrum of the fiber grating, which was initially used to amplify the pump. Erbium-doped fiber amplifier 1 of the laser is now also used as the wide-spectrum light source required by the fiber grating, as shown in Figure 1. An optical circulator is added instead of an optical isolator to one end of the FUT to improve the accuracy of the stress and temperature measurements. This allows the observation of the optical spectra of the FBGs in order to judge the variations in temperature and stress at two specific points.

The scheme of the system is shown in Figure 1. The narrow linewidth distributed feedback (DFB) laser operates at 1550 nm, and Erbium-doped fiber amplifiers (EDFAs) are used to boost the optical signals attenuated by the fiber. Two polarization controllers are used to appropriately control the signal polarization states. The arbitrary waveform generator (AWG) provides an external time pulse to the pulse pattern generator (PPG). A signal generator (SG) can provide 10.5–11.0 GHz continuous wave to the upper electro-optical modulator (EOM1) in order to modulate the probe light, while the PPG is used to provide a pulse signal to the bottom electro-optical modulator (EOM2) to modulate the pump light. The pulse width generated here is closely related to the spatial resolution. The real-time oscilloscope (RTO) is used to conduct the time-domain positioning analysis. The modulator bias controller (MBC) has the function of modulating the bias controller of EOM1. The fiber ratio coupler on the left-hand side is used to split the DFB laser power evenly. Please note that the BOTDA system required two coherent light sources to interact with each other. The built-in optical isolators in optical circulators (OCs) and EDFAs can prevent damage due to reflected amplified optical signals and Rayleigh backscattering. Another function of the OCs is to route the optical signal to the correct direction. The tunable filter can suppress sideband power and improve the optical signal-to-noise ratio (OSNR). In a BOTDA system, the pulse light acts as the sensing probe. When it passes through the optical fiber, a Brillouin gain value is obtained. In Figure 1, the fiber under test (FUT) is presented, including 16 km single-mode fiber (SMF) and two pieces of dispersion-shifted fiber (DSF). An optical circulator (OC) is located on the right-hand side of the FUT. The signal generator is adjusted from 10.5 GHz to 11 GHz at frequency intervals of 0.01 GHz.

## 3. Results and Discussion

### 3.1. 2D and 3D Graphic of Brillouin Frequency

**Figure 2:**
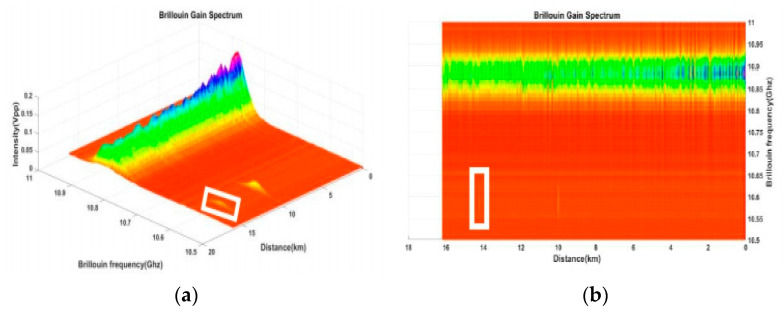
**Analyses of** Brillouin frequency versus fiber distance and optical output power using MATLAB software. (**a**) 3D graphic. (**b**) 2D graphic.

In order to demonstrate the spatial resolution of 2 m over a FUT distance of up to 16 km achievable in the proposed BOTDA system, we added 3 m and 2 m DSFs in the middle of the 16 km optical fiber at room temperature, while the rest of the fiber spool was SMF. The Brillouin gain spectrum (BGS), which takes an average of 300 acquisitions, can be calculated and transformed into 2D and 3D graphics using MATLAB software, as shown in Figure 2a,b. The results were reconstructed for 51 frequencies at measurement intervals of 10 MHz. Frequency drifts at around 10 km were observed and measured. The different fiber cores of SMF and DSF have corresponding Brillouin frequencies of 10.88 GHz and 10.57 GHz, respectively. Please note that DSF has a smaller fiber core than SMF, simulating the shift in the frequency of SMF to 10.57 GHz under specific strain. The pump pulse width was set at 20 ns, corresponding to a spatial resolution of 2 m. The X-axis label is the distance (km), and the Y-axis label is Brillouin frequency (GHz). The oscilloscope was used to observe the BGS at two different frequencies. Here, we aim to analyze the Brillouin frequency drift induced by the fiber core diameter. The spatial resolution was optimized following the pulse width used in this experiment. The spatial resolution was reduced from 3 m to 2 m. The optimal BGS was obtained by adjusting the filter to increase the total fiber distance to 16 km. Two pieces of DSF were observed at 10 km and 14 km from the starting points.

### 3.2. Integration of FBG Sensing in the BOTDA System

**Figure 3:**
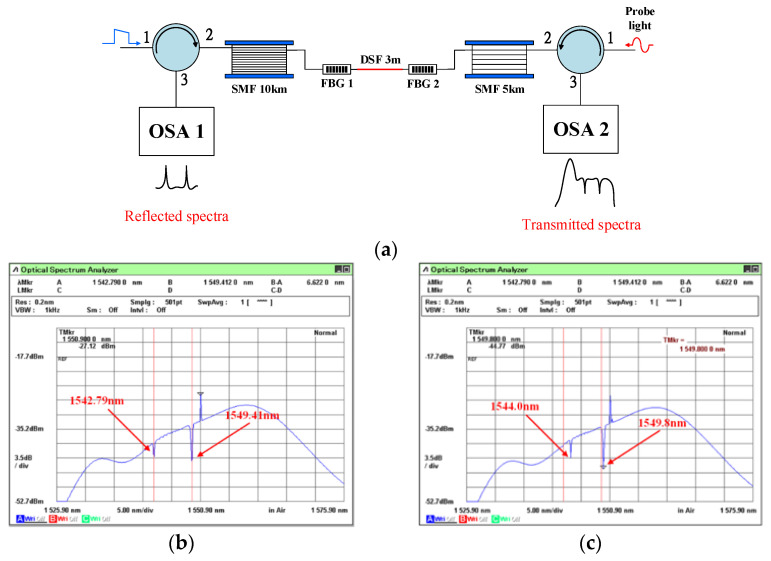
(**a**) Two FBGs are located on both sides of the DSF. (**b**) Before the strain and temperature changes were applied to the FBGs, and (**c**) after the strain and temperature changes were applied to the FBGs.

To assess the impact of temperature on Brillouin frequency drift, a piece of 3 m DSF was immersed in the heater to simulate an increase in temperature. In Figure 3a, two FBGs are deployed between the SMF spools on both sides of the 3 m DSF. Two C band FBGs were made in-house using the standard phase mask method. The length, reflectivity, and 3-dB bandwidth of this FBG were 1.2 cm, about 90%, and 0.25 nm, respectively. The KrF Excimer Laser had a lasing wavelength of 248 nm. A commercial phase mask is used to create a periodic refractive index variation structure.

**Figure 4:**
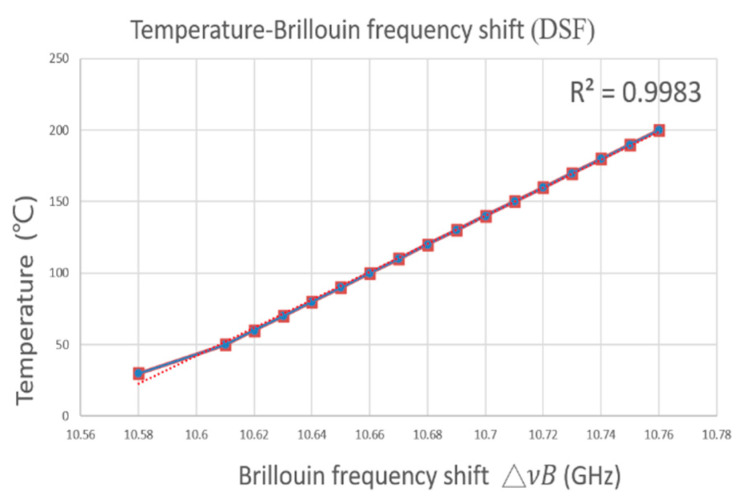
The fiber Brillouin frequency changes due to temperature variation.

FBG1 has an original central wavelength of 1542.79 nm at 25 °C, and FBG2 has an original central wavelength of 1549.41 nm before the application of strain, as shown in Figure 3b. In Figure 3c, the transmitted spectra of FBG1 is shown to be 1544.0 nm at 77 °C, while that of FBG2 is 1549.8 nm; when the strain stage elongates, it moves 0.3 mm, corresponding to 400 με. The measured wavelength variations as a result of temperature and strain are 2311 pm/°C and 0.975 pm/με, respectively, for FBG1 and FBG2. Based on the above experiments, we determined that the BOTDA system integrating FBGs was a good platform for observing both Brillouin frequency drift and the distributed fiber and variation in FBG central wavelengths due to changes in temperature and strain. The temperature against the Brillouin frequency of DSF was measured twice, and the results are shown in Figure 4. We found that the experimental curves were entirely linear, with an R^2^ of 0.9983. These results confirm the feasibility of integrating point-to-point fiber sensing and distributed fiber sensing in a system. Using cost-effective equipment and devices, the sensing resolution was 2 m, compared to the expensive one presented in [21], which had a spatial resolution of 1 m over a 50 km range. Moreover, our hybrid sensing system is built with FBGs in the proposed BOTDA system.

The hybrid system adds FBGs in the fiber under the test (FUT) region. The FBGs measure the temperature and strain change precisely at specific points. This makes it possible to accurately detect disturbances within small ranges, compensating for the limited spatial resolution of BOTDA sensing systems. Additionally, a point-to-point sensing system could solve the dead zone problem. The temperature and strain can therefore be precisely detected and measured.

### 3.3. Reproducibility and Repeatability Test

**Figure 5:**
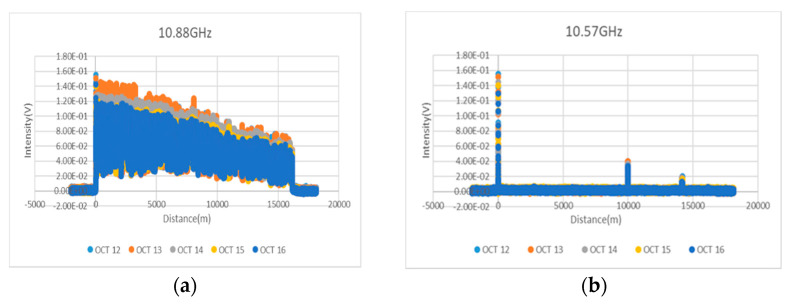

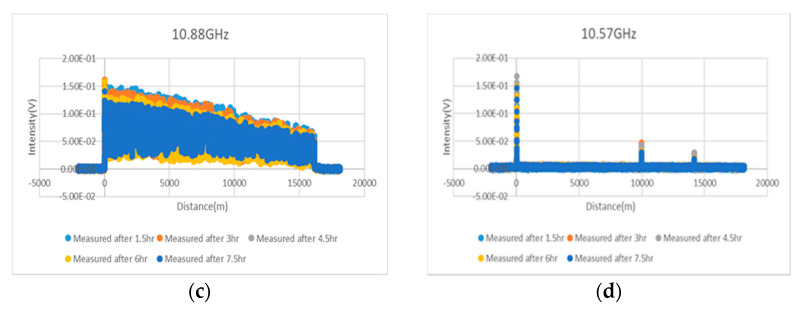
(**a**) The optical intensity against fiber distance data in BOTDA at 10.88 GHz, (**b**) the optical intensity against fiber distance data in BOTDA at 10.57 GHz addressing the reproducibility issue, (**c**) the optical intensity against fiber distance data in BOTDA at 10.88 GHz, (**d**) the optical intensity against fiber distance data in BOTDA at 10.57 GHz addressing the repeatability issue.

In order to address the reproducibility issue, two sections of 2 m DSF were inserted at 10 km and 14 km in the BOTDA system, and the data were measured once every day for five days. A different student measured each day. As a result, the optical intensity against the fiber distance data, which is the frequency intensity at 10.88 GHz against the distributed fiber distance, overlaps very well, as shown in Figure 5a. The overlapping data at a frequency of 10.57 GHz are also shown in Figure 5b. In order to address the repeatability issue, the setup is the same. Two pieces of 2 m DSF were inserted at 10 km and 14 km in the BOTDA system, and the data were measured five times with an interval of 1.5 h by the same person. The data overlap very well, as shown in Figure 5c, which is the frequency intensity at 10.88 GHz against the distributed fiber distance. The overlapping data at a frequency of 10.57 GHz are also shown in Figure 5d. Therefore, we could confirm that the proposed BOTDA system has good performance of reproducibility and repeatability.

The innovation and novelty of this work involve the integration of point-to-point fiber sensing with distributed fiber sensing. That is the use of individual FBGs to test temperature and strain along with a distributed fiber system. Most prior works have only tested the temperature or strain parameters individually. We also suggest that the FBGs should be located at the places most likely to experience temperature/strain variation. The FBGs could also be located in the dead zone of the distributed fiber sensing (DFS) system, where the DFS system cannot detect parameters variation.

## 4. Conclusions

Distributed fiber sensing (DFS) can provide real-time monitoring for various applications. The entire fiber link is the sensing element, but the resolution accuracy is limited. In this paper, distributed fiber sensing and point-by-point FBG sensing were integrated into a single system called a hybrid fiber sensing system for monitoring temperature, pressure, vibration, and strain. However, both the insertion loss and cost increase when more FBGs are added. In this paper, a hybrid fiber sensing scheme was constructed by combining FBGs in DFS. It was constructed by putting several FBGs at certain places where the temperature and strain change in BOTDA fiber systems.The hybrid fiber sensing system has the advantage of all fiber links, and the most dangerous/important locations are sensed precisely. The 16 km BOTDA fiber system has a 2 m spatial resolution overall for temperature and strain monitoring in our work. Furthermore, two FBGs were deployed in the observation zone to improve the temperature and strain sensitivity, corresponding to an FBG shift of 1.21 nm and 0.39 nm, respectively. In summary, the advantages of integrating distributed and point-to-point fiber sensing strategies were demonstrated and analyzed with good results.

## Data Availability

The data presented in this study are available on request from the corresponding author. The data are not publicly available due to privacy restrictions.
